# Special Issue: Cytotoxicity, Antioxidant and Anticancer Activity of Natural Products, 2nd Edition

**DOI:** 10.3390/ijms27041623

**Published:** 2026-02-07

**Authors:** Balik Dzhambazov

**Affiliations:** Department of Developmental Biology, Faculty of Biology, Paisii Hilendarski University of Plovdiv, 24 Tsar Assen Str., 4000 Plovdiv, Bulgaria; balik@uni-plovdiv.bg; Tel.: +359-32-261-535

## 1. Introduction

Natural products have long been used as a source of anticancer agents and continue to attract intense interest as complementary or alternative cancer therapies due to their multi-targeted action and generally lower toxicity compared to synthetic drugs [1]. Historically, natural products and their derivatives have contributed significantly to drug discovery, including the development of first-line anticancer agents, such as paclitaxel, vincristine and doxorubicin [2]. In recent decades, studies on compounds derived from plant, microbial and aquatic organisms have revealed a vast chemical diversity with potential therapeutic effects, including antioxidant and anticancer activities [3]. Such compounds can suppress tumor growth while sparing normal cells. For example, polyphenols such as curcumin, quercetin, genistein and resveratrol are known for their safety and efficacy against various types of cancer [4]. Furthermore, natural compounds often show antioxidant properties and immunomodulatory effects that can influence the tumor microenvironment. Spices such as rosemary, sage, oregano and turmeric can play a dual role—as antioxidants and as anti-inflammatory agents. These dual properties can be therapeutically advantageous—antioxidants may protect normal tissues and reduce chronic oxidative stress (a factor in carcinogenesis), while the prooxidant action of certain natural products can selectively induce cancer cell death via oxidative stress. In fact, many natural compounds induce apoptosis or cell cycle arrest in cancer cells by modulating key pathways related to oxidative homeostasis, inflammation and DNA repair. [4,5]. The use of these natural compounds for effective therapy is restricted by a number of challenges, as many of the isolated bioactive components show only modest efficacy in clinical trials due to poor bioavailability or pharmacokinetic issues [1]. To overcome these limitations, researchers are proposing novel delivery systems, synergistic combinations, and structural modifications of natural molecules. In general, the integration of natural products into cancer therapy involves establishing their direct cytotoxic effects on cancer cells, as well as their antioxidant and immunomodulatory properties, to improve patient outcomes [4].

The first edition of this Special Issue, “Cytotoxicity, Antioxidant and Anticancer Activity of Natural Products” (https://www.mdpi.com/journal/ijms/special_issues/ Antioxidant_Anticancer, accessed on 1 February 2026), illustrated how various natural molecules induce cancer cell death, inhibit metastasis, modulate redox balance and synergize with standard therapies. Whether by inducing apoptosis (rutaecarpine, saponins), disrupting cancer metabolism and signaling (niclosamide, curcumin via let-7C, thymosin β4) or creating insurmountable oxidative stress (ascorbate), these natural compounds exert potent cytotoxic effects on cancer cells while sparing normal cells. Many of them exhibit multiple actions. For example, green tea saponins simultaneously modulate apoptosis, inflammation and angiogenesis, and molecules derived from marine organisms can affect metastasis and cell cycle pathways, suggesting an advantage over drugs with a single mechanism of action. Synergistic effects have also been demonstrated. Natural products can be combined with each other or with standard therapies to achieve synergistic effects, pointing to combination strategies that may improve efficacy and reduce toxicity in patients, for example, polyphenol mixtures that mutually enhance efficacy or ascorbate enhancing the effects of cisplatin.

## 2. An Overview of the Published Articles

This Special Issue (“Cytotoxicity, Antioxidant and Anticancer Activity of Natural Products—2nd Edition”) (https://www.mdpi.com/journal/ijms/special_issues/31UIC4K986, accessed on 1 February 2026) includes primary studies and reviews that illustrate the diverse ways in which natural products and their derivatives exhibit cytotoxic, antioxidant and anticancer activities. Individual contributions range from mechanistic evaluations of individual plant molecules in cancer models to broad analyses elucidating mechanisms of action and novel formulation strategies. An overview of the published articles is provided below, highlighting their key findings and emerging topics in the field.

Kusaczuk et al. (Contribution 1) studied the flavonoids quercetin (QCT) and kaempferol (KMF) in glioblastoma T98G cells. Both flavonoids significantly reduced cell viability and triggered apoptosis by caspase-3/7 and caspase-9 activation. These flavonoids cause oxidative stress and endoplasmic reticulum (ER) stress in cancer cells, leading to increased generation of reactive oxygen species (ROS) and dysregulation of antioxidant enzymes. Authors demonstrated the in vivo efficacy using a chick chorioallantoic membrane (CAM) tumor model, with both compounds reducing tumor growth. QCT and KMF satisfied Lipinski’s Rule of Five and have properties favorable for central nervous system drugs. These findings suggest that naturally occurring flavonoids such as quercetin and kaempferol are plausible candidates for glioblastoma therapy.

Xu et al. (Contribution 2) reviewed the fungus *Omphalia lapidescens*, highlighting its historical use as an adjuvant in clinical cancer treatments in East Asia and identifying polysaccharides, sterols and proteases as active anticancer constituents. Many of the studies reviewed focused on *Omphalia*’s cytotoxicity against cancer cells and its ability to induce apoptosis or cell cycle arrest. The authors point out that detailed investigation of its natural metabolites and their mechanisms of action is an area where further research is required. They provide a valuable reference for future studies aiming to explore *O. lapidescens* as a source of natural anticancer agents. Some polysaccharides from *O. lapidescens* have been reported to modulate the immune system, which could indirectly contribute to anticancer activity.

Araque et al. (Contribution 3) synthesized novel sesquiterpene-aryl derivatives based on drimenol (a natural sesquiterpene) and evaluated their cytotoxic activity against MCF-7 breast cancer cells. Compound ***14c*** showed the highest cytotoxicity toward MCF-7 cancer cells while being significantly less toxic to non-tumorigenic MCF-10 cells (a selectivity index was better than that of the reference drug daunorubicin); ***14c*** induced oxidative stress and activated caspases-3/7, confirming apoptosis induction. It was found to selectively inhibit topoisomerase II activity, with negligible effect on topoisomerase I. The authors conclude that aryl-modified sesquiterpenes such as ***14c*** represent promising anticancer agents, validating the strategy of semisynthetic optimization of natural terpenoids.

Teneva et al. (Contribution 4) explored *Tolypothrix* cyanobacterial strains as a source of bioactive compounds with anticancer, antioxidant, and anti-inflammatory effects. The authors reported dose-dependent cytotoxicity of non-polar *Tolypothrix* extracts against all cancer cell lines tested, with negligible toxicity toward normal cells at equivalent doses. Alongside cytotoxicity, the extracts demonstrated significant antioxidant activity in vitro—free radical-scavenging activity increased with extract concentration in both DPPH and ABTS assays. In addition, the *Tolypothrix* extracts exhibited anti-inflammatory properties, evidenced by a dose-dependent reduction in proinflammatory cytokines (IL-6 and TNF-α) secreted from LPS-stimulated macrophages. Chemical analysis via GC-MS revealed a complex mixture of constituents, including 26 different fatty acids, in the cyanobacterial extracts. Stearidonic acid was identified as a contributor via docking studies, suggesting multi-target therapeutic potential. The study concludes that *Tolypothrix* strains show promising anticancer, antioxidant and anti-inflammatory properties, making them attractive candidates for bioscreening and development of novel natural product-based therapeutics.

Olicheva et al. (Contribution 5) analyzed combinations of dihydroquercetin (taxifolin) with α-tocopherol (vitamin E), finding additive antioxidant effects and prolonged radical-scavenging profiles, offering insights into synergistic formulation. The authors highlight that this insight could guide the rational design of antioxidant formulations in nutraceuticals or pharmaceuticals, ensuring that combinations of natural antioxidants provide prolonged protection against oxidative damage.

Calzada et al. (Contribution 6) tested a panel of eleven acyclic terpenoids for anti-lymphoma activity, using a combination of in vitro, in vivo, and in silico approaches. Citral (C3), geraniol (C4), nerol (C6), and farnesol (C9) exhibited significant anticancer activity and were predicted to target HMG-CoA reductase, a key enzyme in the mevalonate pathway. Overall, this study provides preclinical evidence that simple natural terpenoids have genuine anticancer effects in vivo. The study reinforces the concept that natural products can yield not only new drug candidates but also novel insights into biochemical vulnerabilities of cancer.

Cheng et al. (Contribution 7) fractionated *Polygonum cuspidatum* root extract and identified the ethyl acetate fraction as rich in polyphenols and flavonoids. This fraction showed potent antioxidant, enzyme-inhibitory and antibacterial activities. Via HPLC-PDA and GC-MS analysis, tannic acid (hydrolyzable tannin) and emodin (anthraquinone) were identified as the main constituents of this bioactive fraction. The authors conclude that the polyphenol-rich *P. cuspidatum* fraction, especially the EtOAc fraction, is a promising natural source of multi-functional bioactive agents for use in the food, pharmaceutical, and cosmetic industries.

Moura et al. (Contribution 8) provided a comprehensive review of carnosic acid, a diterpene from rosemary, and its semisynthetic derivatives as anticancer agents. Carnosic acid is a well-known natural antioxidant (found in *Rosmarinus officinalis* and *Salvia* species) with the ability to modulate cell proliferation, apoptosis and oxidative stress pathways in cancer models. The review discusses how adding or altering functional groups (at C-20 or the aromatic ring of carnosic acid) can improve cytotoxic efficacy, sometimes by increasing proapoptotic activity or by achieving better drug-like properties. Authors found that structural modifications significantly improved anticancer efficacy and selectivity, highlighting the role of medicinal chemistry in enhancing natural product-based therapies.

## 3. Conclusions

The studies published in this Special Issue demonstrate the diversity of mechanisms by which natural products exert cytotoxic, antioxidant, and anticancer effects (Table 1). From inducing oxidative and endoplasmic reticulum stress in cancer cells to modulating enzyme activity and metabolic pathways, these compounds provide a wide range of tools for drug development.

In summary, natural products exert anticancer effects in three interconnected ways:

- Direct cytotoxicity, via apoptosis induction, cell cycle arrest and metabolic disruption;
- Redox modulation, characterized by selective oxidative stress in cancer cells and antioxidant protection of normal cells;
- Tumor microenvironment targeting, including inhibition of angiogenesis, metastasis, inflammation, fibrosis and immune evasion.

Combinations and semisynthetic modifications further enhance their therapeutic potential. These findings support the need for continued investment in research, formulation, and clinical applications in natural products.

## Figures and Tables

**Table 1:** Overview of natural products with cytotoxic, antioxidant and anticancer activity in the Special Issue “Cytotoxicity, Antioxidant and Anticancer Activity of Natural Products”.

Natural Product	Source	Cancer Models	Molecular Mechanisms	Effects
Rutaecarpine	*Evodia rutaecarpa* (plant alkaloid)	Esophageal squamous carcinoma (in vitro, xenograft)	G_2_/M arrest, p53–Bax upregulation, caspase-9/3 activation	Tumor growth inhibition in vivo, limited toxicity to normal tissue
Polyphenols (curcumin, genistein, quercetin, resveratrol)	Dietary phytochemicals	Acute lymphoblastic leukemia	Mitochondrial dysfunction, membrane permeability changes, apoptosis	Strong synergy at physiologically relevant doses, sparing of normal fibroblasts
Curcumin	*Curcuma longa*	Renal carcinoma (TRAIL-resistant)	let-7C miRNA upregulation, CDK/cyclin suppression, metabolic reprogramming	Sensitizes resistant tumors to apoptosis
Niclosamide	Salicylanilide derivative	Osteosarcoma	TGFBI suppression, ERK1/2 inhibition	Potent anti-migratory and anti-invasive effects
Emodin/aloe-emodin (PDT)	Plant anthraquinones	Melanoma, squamous carcinoma	ROS generation, apoptotic vs. necrotic balance	Cancer cell phototoxicity, minimal damage to keratinocytes
Green tea saponins	*Camellia sinensis*	Liver, colon carcinoma, endothelial cells	Caspase-3/BAX activation, PI3K–AKT–VEGFR2 inhibition, NRF2 activation	Anti-angiogenic, anti-inflammatory, antioxidant
Xylooligosaccharides (XOS)	Hemicellulose-derived oligosaccharides	Lung, colon, lymphoma	Glutathione depletion, GR inhibition, lysosomal stress	Tumor-selective redox imbalance, TLR4-mediated anti-inflammatory effects
Ascorbic acid (high dose)	Vitamin C	Oral squamous carcinoma	Mitochondrial ROS induction, redox collapse	Synergistic with cisplatin, minimal synergy in normal cells
Thymosin β4	Endogenous peptide	IPF-associated lung cancer	JAK2/STAT3 inhibition, fibroblast–tumor crosstalk suppression	Anti-fibrotic and anti-tumor dual action
Marine natural products	Algae, sponges, fungi	Colorectal, pancreatic cancers	Apoptosis, thioredoxin inhibition, immune modulation	Novel mechanisms for therapy-resistant tumors
